# Biomechanical comparison of intramedullary nail and plate osteosynthesis for extra-articular proximal tibial fractures with segmental bone defect

**DOI:** 10.3389/fbioe.2023.1099241

**Published:** 2023-03-03

**Authors:** Weihang Gao, Ke Zhao, Yuanyuan Guo, Mao Xie, Xiaobo Feng, Ping Liu, Xin Xie, Dehao Fu

**Affiliations:** ^1^ Department of Orthopaedics, Shanghai Sixth People’s Hospital Affiliated to Shanghai Jiao Tong University School of Medicine, Shanghai, China; ^2^ Department of Orthopaedics, Liyuan Hospital, Tongji Medical College, Huazhong University of Science and Technology, Wuhan, China; ^3^ Department of Pharmacy, Liyuan Hospital, Tongji Medical College, Huazhong University of Science and Technology, Wuhan, China; ^4^ Department of Orthopaedics, Union Hospital, Tongji Medical College, Huazhong University of Science and Technology, Wuhan, China

**Keywords:** proximal tibial fracture, comminuted tibial fractures, intramedullary nailing, locking plate, biomechanical study

## Abstract

**Purpose:** Proximal tibial fractures are common, but the current available internal fixation strategies remain debatable, especially for comminuted fractures. This study aimed to compare the biomechanical stability of three internal fixation strategies for extra-articular comminuted proximal tibial fractures.

**Methods:** A total of 90 synthetic tibiae models of simulated proximal tibial fractures with segmental bone defects were randomly divided into three groups: Single lateral plating (LP), double plating (DP) and intramedullary nailing (IN). Based on the different number of fixed screws, the above three groups were further divided into nine subgroups and subjected to axial compression, cyclic loading and static torsional testing.

**Results:** The subgroup of intramedullary nailing with five proximal interlocking screws showed the highest axial stiffness of 384.36 ± 35.00 N/mm. The LP group obtained the lowest axial stiffness performance with a value of 96.59 ± 16.14 N/mm. As expected, the DP group offered significantly greater biomechanical stability than the LP group, with mean static axial stiffness and mean torque increasing by approximately 200% and 50%, respectively. According to static torsional experiments, the maximum torque of the DP subgroup was 3,308.32 ± 286.21 N mm, which outperformed all other groups in terms of torsional characteristics.

**Conclusion:** Utilizing more than four distal screws did not provide improved biomechanical stability in the LP or DP groups, while a substantial increase in the biomechanical stability of DP was obtained when an additional medial plate was used. For the intramedullary nailing group, increasing the number of proximal interlocking screws could significantly improve biomechanical stability, and the intramedullary nailing with three proximal interlocking screws had similar static and cyclic stiffness as the DP group. The intramedullary nailing with five proximal screws had better axial stability, whereas DP had better torsional stability.

## Introduction

Extra-articular proximal tibial fractures, also known as proximal third tibia fractures, account for 5%–11% of all tibial fractures (Court-Brown and McBirnie, 1995; Buckley et al., 2011; Lowe et al., 2011). The management of such fractures is very challenging given the complexity of the proximal tibia’s anatomical structure and the high demand for the reduction of major weight-bearing bones (Lembcke et al., 2001; Vestergaard et al., 2020; Shen et al., 2021). When the extra-articular proximal tibia is severely comminuted, a treatment utilizing low mechanical strength may fail to fix fracture sites, leading to looseness and breaking of implants and screws (Gosling et al., 2005; Janssen et al., 2007; Demirtas et al., 2019). In order to reduce failure or other complications, it is necessary to achieve a good reduction and rigid fixation of the comminuted fracture, allowing for early mobilization and functional exercise after surgery (Schütz et al., 2005).

The single lateral plate (LP), double plate (DP) and intramedullary nailing (IN) are the common internal fixation modalities for tibial fractures (Haidukewych et al., 2008; Kandemir et al., 2017), but the literature is inconclusive about what approach is best (Vallier et al., 2011; Lee et al., 2014). Previous finite element simulations showed that DP fixation had the best biomechanical stability compared to IN, and static axial stabilization was proportional to the number of distal screws and the length of the locking plate (Chen et al., 2018). When the distal screws of DP were gradually increased from four to eight, stepwise raise in the biomechanical stability was detected. However, other scholars reached different conclusions, showing that IN was superior to LP and DP fixation techniques in terms of static and cyclic compression properties (Feng et al., 2012; Lee et al., 2014). The inconsistent results may be caused by the different bone materials or parametric experimentation used. Finite element simulations also have their inherent shortcoming, which the materials of the cortical and cancellous bone were both simulated and probably did not reflect the actual conditions. Currently, most of the finite element simulations of bone are limited to static loading conditions, which may not adequately account for complicated loading that may occur in activities, because the dynamic imitation will require considerable computer resources and time (Pakdel et al., 2016; Chen et al., 2018). Biomechanical experiments are a more natural and realistic way to simulate complex physiological conditions, including cyclic loading and torsional testing.

To date, there are no reports regarding the comprehensive and systematic comparison of the biomechanical stability of LP, DP and IN fixation techniques in the literature. The optimal number of distal screws for a locking plate and the proximal interlocking screws for intramedullary nailing have long been debated. To that end, the present study investigated the biomechanical properties of three internal fixation strategies in combination with different numbers of screws using an extra-articular proximal tibial fracture model with segmental bone defects.

## Materials and methods

### Specimens and study groups

**FIGURE 1:**
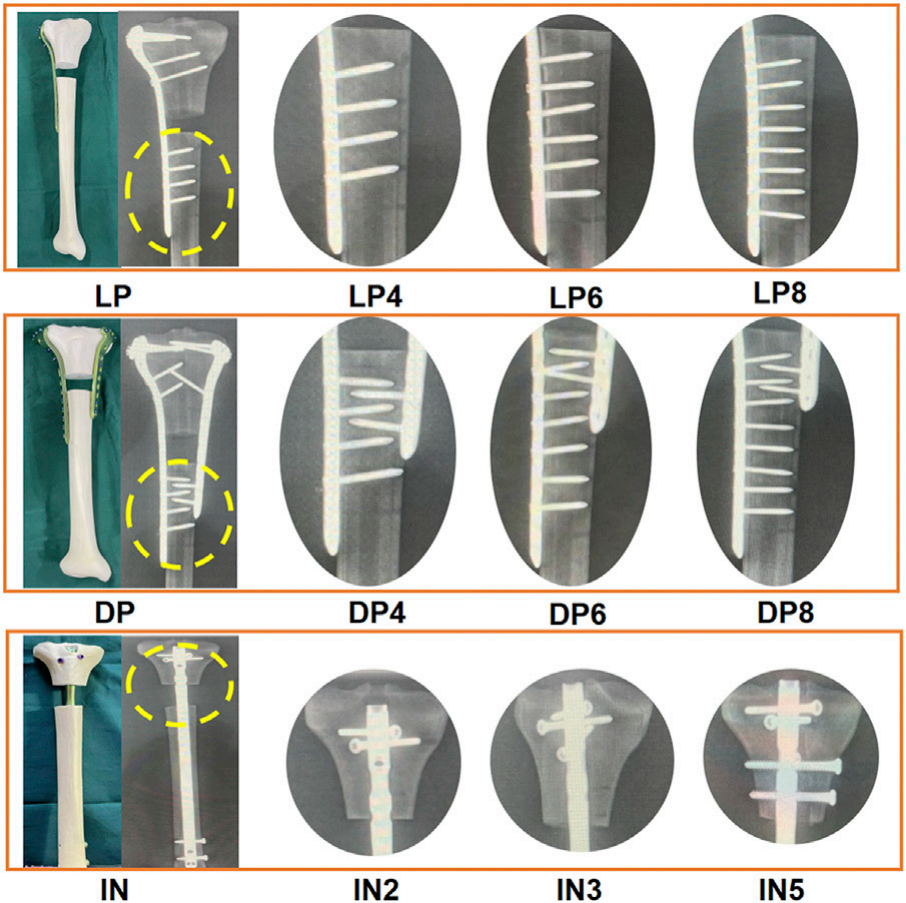
Photographs and radiographic images depicting the nine subgroups that utilized various fixation strategies. Lateral plate fixation group (LP): four distal locking screws (LP4), six distal locking screws (LP6), and eight distal locking screws (LP8). Double plate fixation group (DP): Four distal locking screws of the lateral plate (DP4), six distal locking screws of the lateral plate (DP6), and eight distal locking screws of the lateral plate (DP8). Intramedullary nailing group (IN): Two proximal interlocking screws (IN2), three proximal interlocking screws (IN3), five proximal interlocking screws (IN5).

The current study used a total of 90 synthetic composite tibiae (type 1,110. SYNBONE AG, Malans, Switzerland). Three types of implants [locking compression plate for lateral proximal tibia, locking compression plate for medial proximal tibia, and intramedullary nail (Double Medical Ltd., Xiamen, China)] were used in the study. Both lateral and medial proximal tibial locking plates were anatomically pre-contoured plates. All nails and plates were made of titanium alloys. An extra-articular proximal tibial segmental bone defect model was established in all specimens by templating cut lines on the synthetic bone at 70 mm and 90 mm from the medial tibial plateau to simulate the comminuted proximal tibial fractures (OTA type 41-A3.3) (Kandemir et al., 2017). A two cm synthetic bone fragment was removed without locking screw implantation in the defective zones (Figure 1). An experienced orthopedic surgeon performed all geometric measurements and established the fracture model. Finally, to rule out any damage resulting from implantation, a radiological examination of all the tibiae was conducted to confirm the exact position of the final implantation.

LP fixation group: The 3.5-mm L-shaped lateral plates with different numbers of distal locking screws were categorized into three subgroups (*n* = 10). The six proximal locking screws were identical for all three subgroups, with only the number of distal locking screws varying. The LP4 (176 mm in length), LP6 (203 mm in length) and LP8 (228 mm in length) subgroups received four, six and eight distal locking screws, respectively. Distal fixation was achieved by using 26 mm–28 mm bicortical screws.

DP fixation group: Three subgroups were established for models that used the double plate fixation strategy (*n* = 10). The T-shaped medial plate (135 mm in length) and nine proximal locking screws were identical for all three subgroups, with only the number of distal locking screws differing. The DP4, DP6 and DP8 subgroups received four, six and eight distal locking screws, respectively. Distal fixation was achieved by using 26 mm–28 mm bicortical screws.

IN fixation group: Three subgroups were established for models that utilized intramedullary nailing (320 mm in length, 10 mm in diameter) (*n* = 10). The two distal locking screws were identical for all three subgroups, varying only in the number of proximal interlocking screws. The IN2, IN3 and IN5 groups were fixed using two, three and five proximal interlocking screws, respectively (Figure 1). The size of the screws was 5.0-mm diameter and 50–60 mm in length.

### Experimental procedure

**FIGURE 2:**
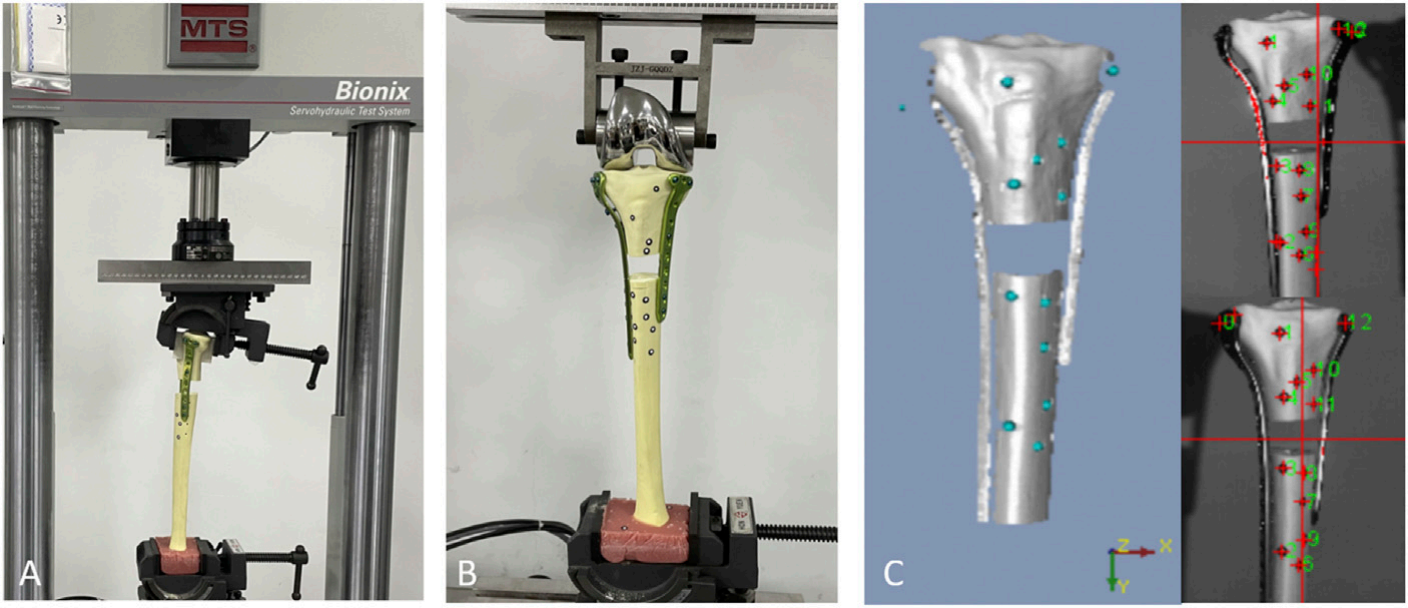
A biomechanical machine was used for torsional testing, **(A)** and a 3D-printed mold was used to fix the proximal fragment. **(B)** In the axial compression and cyclic loading test, the load was applied axially through the femoral component of a knee prosthesis. **(C)** The test setup showed the position of the optical measurement system markers.

The synthetic tibias were potted distally in high-strength resin (Denture Base Materials) and mounted within the loading axis of a material-testing machine (MTS Bionix Servo-hydraulics Test Systems Model 370.02; MTS Systems) using custom-designed alignment fixtures that provided a consistent and repeatable orientation of the repaired tibiae in the loading frame (Figure 2B).

Each subgroup incorporated ten samples; five samples were used for axial compression and static torsional testing, while the other five samples were used for cyclic loading tests. In the axial compression and cyclic loading tests, the load was applied axially through the femoral component of a knee prosthesis. The pre-prepared 3D-printed clamping device was used in the static torsional test to clamp the proximal tibial fragment and facilitate a constant compressive gripping force (Figure 2A). An optoelectrical device with an accuracy of 0.01 mm was used to measure fracture gap movement and fragment displacement (KSCAN-Magic Composite 3D Scanner) (Figure 2C).

Axial compression test: The specimen was loaded with axial loading pressure starting from 0 N to 800 N, with a rate of 50 mm/min. Four load levels with a peak force of up to 200 N, 400 N, 600 N, and 800 N were adapted from previously reported data of the physiological compressive load on an adult knee during a single-limb stance (Ratcliff et al., 2007).

Cyclic loading test: The axial cyclic load was gradually loaded starting from 0 N and finishing at 400 N, at a frequency of 3 Hz for 10,000 cycles (Kandemir et al., 2017; Nie et al., 2020). Displacement was defined as the difference in the crosshead position from the peak of the first cycle to the peak of cycles 2,500, 5,000, 7,500, and 10,000.

Static torsional test: Each specimen was fixed on the mechanical testing machine, and the proximal fragment was able to rotate clockwise with the helical blade as the axis on the plane of the tibial fracture site. A 100 N preload was applied to the tibial plateau to maintain a stable of the tibial axial direction. Then torque was used at a load rate of 2°C/min from 0°C to 5°C.

### Statistical analysis

All the data were represented using mean ± SD. Two-way analysis of variance (ANOVA) was used to compare three or more groups, while Student’s *t*-test was used for two groups comparisons. All statistical analyses were performed using SPSS version 23.0 (IBM, Armonk, New York, United States). Graphs were generated using Graphpad Prism 8.0 (Graphpad Software Inc.). A *p*-value less than 0.05 was considered statistically significant.

## Results

**FIGURE 3:**
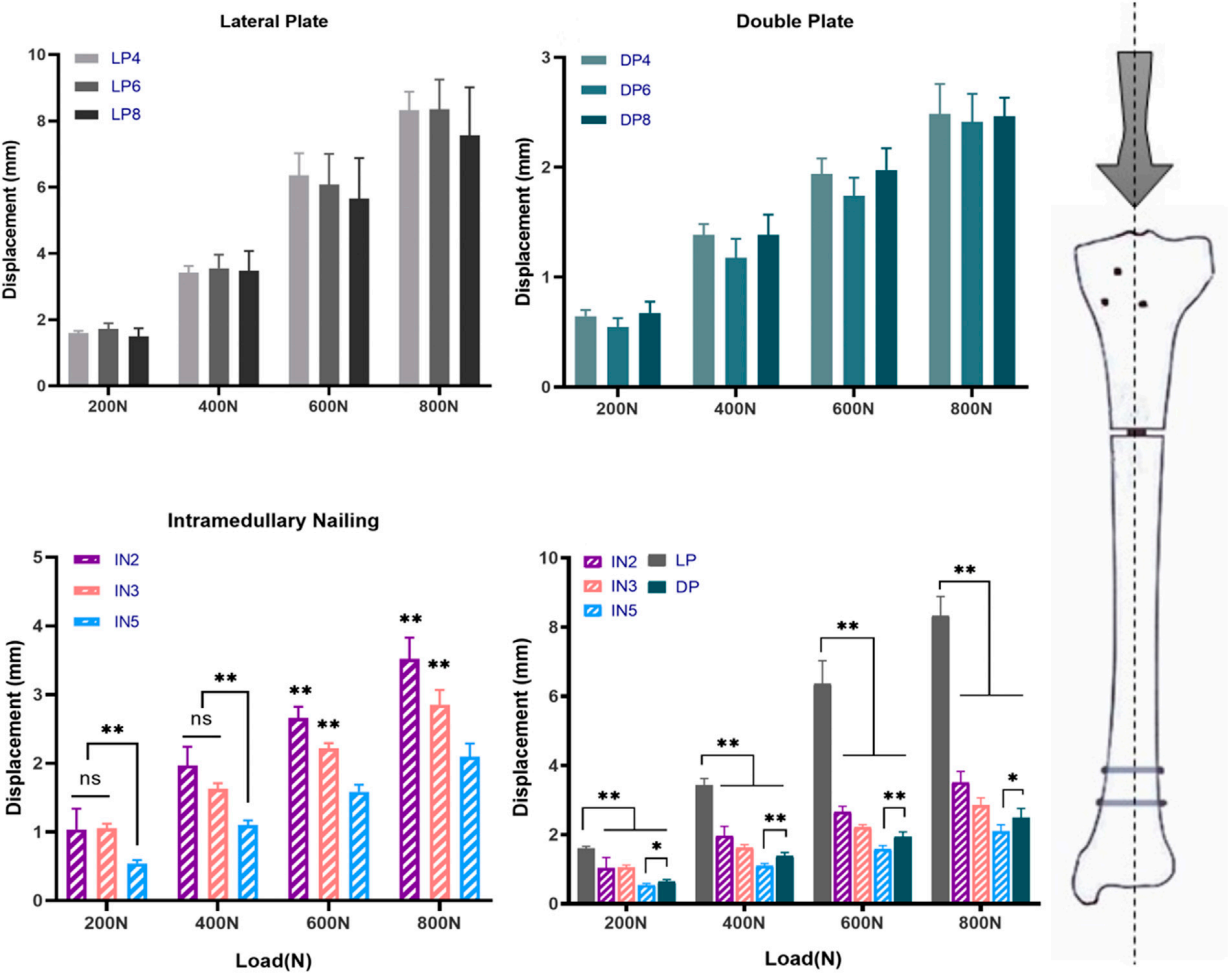
The different fixation modalities: lateral plate fixation groups (LP), double plate fixation groups (DP), intramedullary nailing groups (IN) and multiple comparisons. Values represent the mean axial displacements (measured in mm). (ns, no significant difference; *p < 0.05; **p < 0.01).

**FIGURE 4:**
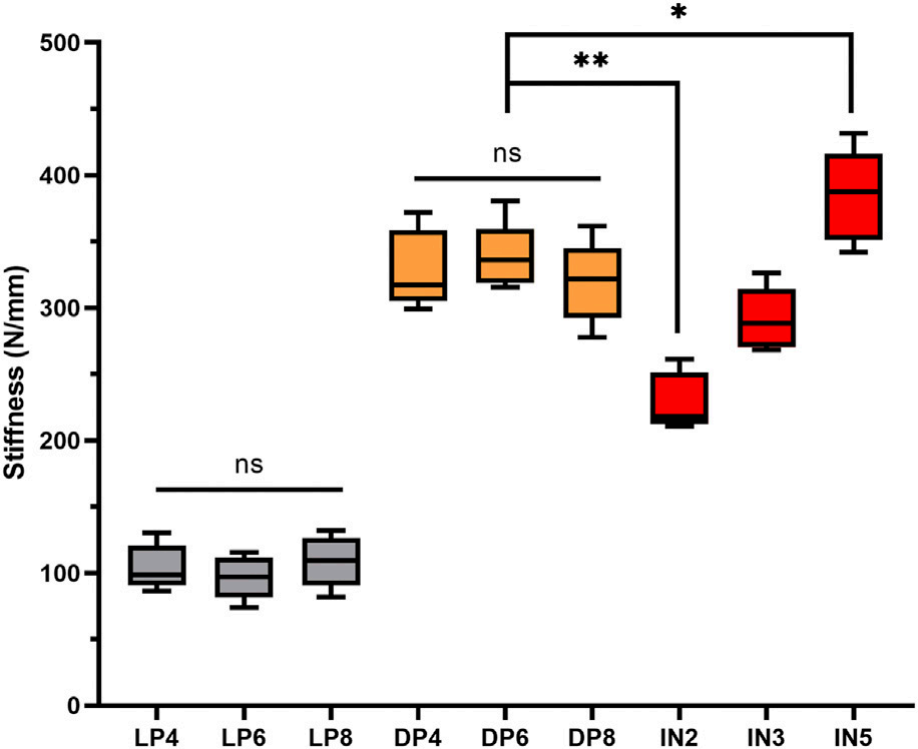
Axial static stiffness (median, range, *n* = 5) and significant differences among groups: lateral plate fixation groups (LP), double plate fixation groups (DP), intramedullary nailing groups (IN). (ns, no significant difference; *p < 0.05; **p < 0.01).

Throughout testing (including axial compression, cycle loading and static torsional testing), loading was continued unless implant-bone construction failure (plate loosening or deformation, or screw loosening or breakage) was detected. There were some small displacements during the reduction process, but the actual difference is very small and does not affect the distribution of screws, so the impact on mechanical stability is also negligible. Statistical analysis of axial stiffness, cycling stability and torsional properties under the same conditions found no significant difference between LP4, LP6 and LP8 subgroups (*p* > 0.05) and between the DP4, DP6 and DP8 subgroups (*p* > 0.05) (Figure 3). As expected, the DP group offered significantly greater biomechanical stability than the LP group, with mean static axial stiffness and mean torque increasing by approximately 200% and 50%, respectively (Table 1). The IN5 subgroup showed the highest axial stiffness of 384.36 ± 35.00 N/mm. The LP group obtained the lowest axial stiffness performance with a value of 96.59 ± 16.14 N/mm. The static axial stiffness values in descending order are as follows: IN5 subgroup > DP group > IN3 subgroup > IN2 subgroup > LP group (Figure 4).

**TABLE 1:** Mean stiffness in N/mm of each group and standard deviation.

Group		Static loading		Cyclic loading	
		Mean	Median (min to max)	Mean	Median (min to max)
Lateral plate (LP)	LP4	104.33 ± 16.99	98.83 (86.46–130.14)	70.77 ± 2.54	70.99 (67.01–73.93)
	LP6	96.59 ± 16.14	96.92 (73.98–115.44)	71.06 ± 3.55	72.54 (65.03–74.18)
	LP8	108.61 ± 19.39	109.42 (81.79–132.01)	80.53 ± 9.55	77.88 (70.25–93.35)
Double plate (DP)	DP4	328.77 ± 25.35	316.96 (298.94–372.09)	137.10 ± 9.67	135.92 (126.18–152.79)
	DP6	338.39 ± 25.35	336.13 (315.33–380.43)	148.50 ± 11.88	147.71 (136.52–162.60)
	DP8	319.25 ± 30.54	321.67 (277.73–361.34)	141.50 ± 17.53	133.91 (125.47–169.78)
Intramedullary nailing (IN)	IN2	229.01 ± 21.41	217.86 (210.58–261.01)	131.58 ± 15.83	124.61 (116.72–156.68)
	IN3	291.43 ± 23.62	288.18 (268.19–326.04)	163.55 ± 16.81	164.81 (137.79–181.82)
	IN5	384.36 ± 35.00	387.60 (341.88–431.73)	190.19 ± 17.12	186.74 (167.93–213.90)

**FIGURE 5:**
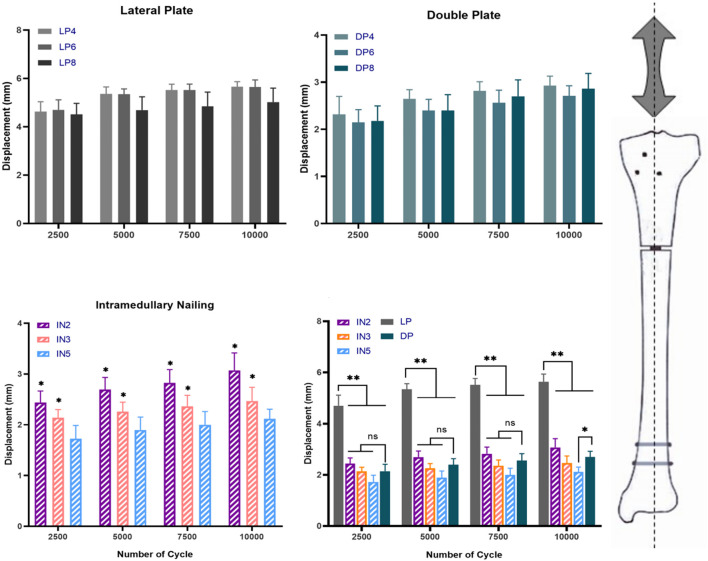
The different fixation modalities: lateral plate fixation groups (LP), double plate fixation groups (DP), intramedullary nailing groups (IN) and multiple comparisons. Values represent the mean axial displacement of the proximal tibial fragment after 10,000 cycles under 400 N axial loads. (measured in mm). (ns, no significant difference; *p < 0.05; **p < 0.01).

**FIGURE 6:**
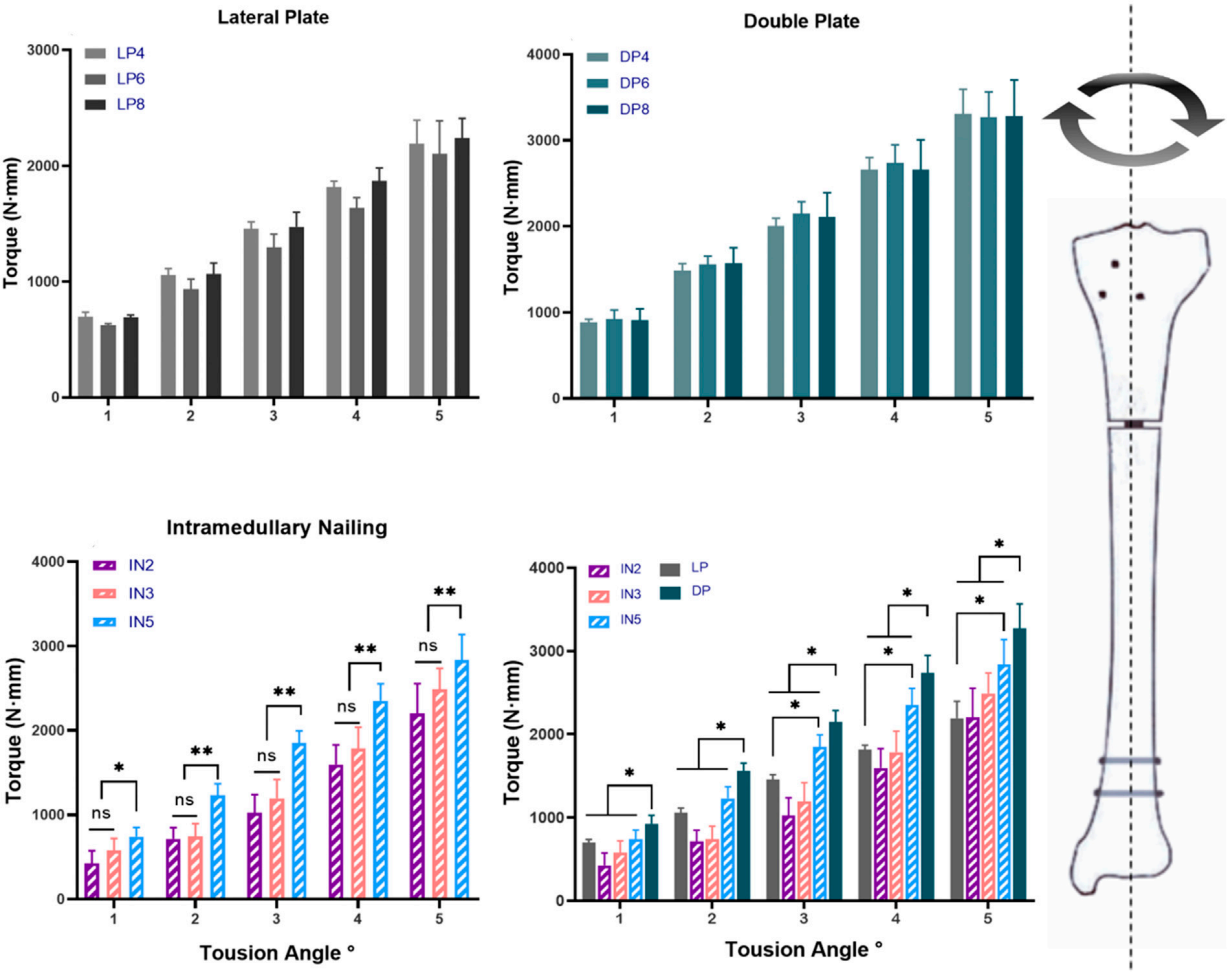
Torque and the torsion angle relationship of different models: lateral plate fixation groups (LP), double plate fixation groups (DP), intramedullary nailing groups (IN) and multiple comparisons. (ns, no significant difference; *p < 0.05; **p < 0.01).

In the cyclic loading test, the displacement of the DP group in each cycle of the nodes was less than that of the IN2 subgroup (*p* > 0.05) but greater than that of the IN5 subgroup (*p* < 0.05). Compared to the LP group, the median stiffness values of the DP group improved by approximately 110%, while the IN5, IN3 and IN2 subgroups increased by around 160%, 120%, and 80%, respectively (Figure 5). In the static torsional test, the maximum torque of the DP subgroup was 3,308.32 ± 286.21 N mm, while that of the IN5 subgroup was 2,836.54 ± 300.84 N mm. Compared to the IN5 subgroup, the torsional characteristics of DP were boosted by more than 15% (Figure 6).

## Discussion

The intramedullary nailing and locking plate fixation have offered an alternative to treating proximal tibial fractures (Haidukewych et al., 2008; Kandemir et al., 2017). In recent years, a growing body of research has focused on improving the stability of internal fixation, as well as LP, DP, and IN fixation modalities (Freeman et al., 2011; Kandemir et al., 2017). Although several studies compared the stability of different internal fixations for extra-articular proximal tibial fractures, the conclusions were inconsistent. Some researchers have advocated the use of DP fixation on account of bilateral mechanical advantages, while others have considered LP and IN fixations as minimally invasive options that could provide acceptable biomechanical stability (Feng et al., 2012; Högel et al., 2013; Kandemir et al., 2017). Moreover, the number of screws required, not only for the locking plate but also for the intramedullary nail, is still controversial. Abundant studies have recommended that at least three screws should be inserted on either side of the fracture in each main fragment; adding a fourth screw had little effect on axial stability but could improve torsional stability (Hertel et al., 2001; Gautier and Sommer, 2003; Cronier et al., 2010). Four screws are commonly used on either side of comminuted fractures in trauma surgery, but it is unclear whether further increasing distal screws would enhance biomechanical stability. Therefore, further comprehensive and systematic experiments with uniformly standardized fracture models and experimental parameters are required to establish a firm conclusion.

Our results showed that biomechanical stability was significantly lower in the LP group compared to the DP and IN groups, which could be explained by the LP belonging to the eccentric load carriers (Mueller et al., 2005). Additionally, our investigation revealed that the mean static stiffness increased about two-fold when a medial assisting plate was added. Similar results obtained from Lee et al. (2014) showed that double plating provided greater biomechanical stability than single-lateral plating, increasing by approximately 17.5%. Moreover, when the number of distal screws exceeded four, no obvious increase of the biomechanical stability was observed. This phenomenon could be explained by stress being concentrated in the region between the fracture gaps and strain being concentrated primarily in the near screw of the fracture line (Cronier et al., 2010; Chen et al., 2018; Travascio et al., 2021). Taken together, we showed that double plating construct might be superior to LP fixation for treating proximal tibial fractures with segmental defect and no more than four distal locking screws recommended.

According to the present study, the IN5 subgroup had the highest biomechanical stability among the intramedullary nail groups, while the IN2 subgroup displayed the lowest. This would suggest that increasing proximal interlocking screws from two to five would significantly improve biomechanical stability when the intramedullary nail is used to fix tibial proximal fracture. Consistent with a previous report (Hansen et al., 2007; Horn et al., 2009; Gkouvas et al., 2019), the maximum number of proximal interlocking screws in all possible directions should be used to achieve maximum axial stability for intramedullary nailing. The DP group had lower static and cyclic stiffness than the IN5 subgroup, but significantly higher than that of the LP and IN2 groups and was comparable to the IN3 group. This result indicates that at least three proximal interlocking screws are required for IN to achieve the same axial stability as the DP group. Next, the torsional properties of the different internal fixation methods were further investigated in our study, as shown in Figure 6. The results revealed that the DP group showed the highest torsional properties compared to the IN and LP groups. The tibia was not only subjected to axial pressure from the knee joint, but internal and external rotation were also essential movements in routine activities. Double plating with more torsional resistance would provide more stability and safety during torsional movements. In terms of biomechanics, IN and DP fixations primarily provide axial and lateral support, respectively. The IN fixation method provided more axial stiffness for construct stability, whereas DP could handle torsional loads better. Although the IN technique has higher axial stiffness and less soft tissue injury, it could lead to the loss of proximal fragment fixation (Stedtfeld et al., 2004; Vallier et al., 2011). Considering the characteristics of DP fixation, which distributes stress more evenly, it might be more suitable to treat non-osteoporotic young patients who tend to fast recovery and whose bones are biomechanically sturdy. These biomechanical results did not recommend single lateral locking plates as the optimal fixation modality for patients with proximal tibial segmental defect or comminuted fracture due to the weak axial and torsional stability.

Nevertheless, it is undeniable that this study has some inherent limitations. Artificial bone models could not completely simulate the biological changes that occur *in vivo* because the soft tissues around the knee joint and the role of the fibula were not taken into account. Compared with cadaveric *in vitro* studies, the artificial bone models did not account for the physiological variations in density and distribution of force in the human bone.

## Conclusion

Our study came to several conclusions based on the above findings. First, using more than four distal screws in the locking plate (LP and DP) did not improve biomechanical stability, which is completely different from the finite element mechanical analysis. Second, the biomechanical stability of IN was improved as the proximal interlocking screws were increased. At least IN with three proximal interlocking screws is recommended to achieve sufficient stability, which has similar static and cyclic stiffness as the DP group. Third, the single lateral plate displayed relatively low stability and should be used with caution in patients with proximal tibial segmental defect or comminuted fractures. Instead of the DP always providing optimal biomechanical properties as in previous finite element analysis, our results showed better axial stability in IN5 and better torsional stability in DP fixation strategies.

## Data Availability

The original contributions presented in the study are included in the article/Supplementary Material, further inquiries can be directed to the corresponding authors.
